# Retrospective Observational Study and Analysis of Two Different Photobiomodulation Therapy Protocols Combined with Rehabilitation Therapy as Therapeutic Interventions for Canine Degenerative Myelopathy

**DOI:** 10.1089/photob.2019.4723

**Published:** 2020-04-16

**Authors:** Lisa A. Miller, Debbie (Gross) Torraca, Luis De Taboada

**Affiliations:** ^1^ Companion Animal Health, LiteCure LLC, New Castle, Delaware, USA.; ^2^ Wizard of Paws Physical Rehabilitation for Animals, Colchester, Connecticut, USA.

**Keywords:** degenerative myelopathy, photobiomodulation, low-level laser therapy, LLLT, ALS, amyotrophic lateral sclerosis, canine, physical rehabilitation

## Abstract

***Objective:*** The objective of this retrospective review was to examine the impact that adding photobiomodulation therapy (PBMt) to rehabilitation therapy had on the pathology of degenerative myelopathy (DM) in canine patients.

***Background:*** Canine DM is a progressive, fatal neurodegenerative disease for which there exists a dearth of effective treatments, limiting clinicians to pursue symptom palliation.

***Methods:*** Clinical records of dogs referred for presumed DM to a specialty rehabilitation facility were screened for patients meeting study criteria. Qualifying patients were divided into two groups: Protocol A (PTCL-A) and Protocol B (PTCL-B) group, based on the PBMt protocol used. Data related to demographics, diagnostics, rehabilitation protocols, and progression of clinical signs were collected. Data were analyzed to determine differences in outcomes between the two treated groups and historical data expectations, as given by a previously published study.

***Results:*** The times between symptom onset and euthanasia of dogs in the PTCL-B group: 38.2 ± 14.67 months (mean ± SD), were significantly longer than those of dogs in the PTCL-A group: 11.09 ± 2.68 months. Similarly, the times between symptom onset and nonambulatory paresis (NAP) or paralysis of dogs in the PTCL-B group: 31.76 ± 12.53 months, were significantly longer than those of dogs in the PTCL-A group: 8.79 ± 1.60 months. Further, Kaplan–Meier survival analysis showed that the times from symptom onset to NAP of dogs in the PTCL-B group were significantly longer than those of dogs in the PTCL-A group (Mantel-Cox Log Rank statistic = 20.434, *p* < 0.05) or the historical data group (Mantel-Cox Log Rank statistic = 16.334, *p* < 0.05).

***Conclusions:*** The data reviewed show significantly slower disease progression—longer survival times—for patients in the PTCL-B group than those in the PTCL-A group or published historical data. Further studies are warranted.

## Introduction

Canine degenerative myelopathy (DM) is a progressive adult-onset neurodegenerative disease^1^ characterized by progressive generalized proprioceptive ataxia of the pelvic limbs, asymmetric upper motor neuron (UMN) paraparesis, and a lack of paraspinal hyperesthesia, which progresses to lower motor neuron (LMN) paralysis of the pelvic limbs, and, eventually, the thoracic limbs.^2^ Sharing similarities in cause, clinical signs, and disease progression to some forms of amyotrophic lateral sclerosis (ALS) in humans, DM is a naturally occurring animal model for this disease. Mutations in the superoxide dismutase 1 (SOD1) gene and subsequent conformational alterations to the SOD1 protein and associated toxicity are responsible for 20% of genetic ALS and most cases of canine DM.^3^ Although the German Shepherd Dog is the most commonly affected breed, DM is now known to occur in multiple breeds, with an overall prevalence of 0.19%.^4^

ALS and DM are progressive, incurable, fatal diseases; thus, clinicians look to available and novel therapies, and clinical interventions to improve the overall quality of life and, hopefully, increase the life expectancy of affected patients. However, while ALS patients may be medically managed for relatively long periods of time while their disease progresses and they become severely debilitated—usually dying from respiratory failure within 3–5 years of developing symptoms, dogs with DM are usually euthanized when they become nonambulatory and/or fecal or urinary incontinent (which has been noted at various clinical stages of disease,^2,5^ though usually occurs with paraplegia), both of which present challenges to at-home care by pet owners.

Canine DM symptom progression is consistent across and within breeds, with a mean age of symptom onset of 9 years of age,^2^ though younger dogs have been affected,^6^ and there are four generally recognized,^2^ distinct stages of disease progression:

I . general proprioceptive ataxia with spastic UMN paraparesis,
I I. nonambulatory paraparesis to paraplegia,
I II. LMN paraplegia to thoracic limb paraparesis,
I V. LMN tetraplegia and brain stem dysfunction

Time of progression from stage I to stage II is generally 6–9 months.^2^

Various therapeutic protocols have been tried as interventions in attempts to slow DM's disease progression and/or for symptom palliation; none have been significantly successful.^7,8^ Only daily intensive physiotherapy has demonstrated some benefit as a DM supportive therapy—DM patients who received intensive daily physiotherapy (defined in the study as gait exercise at least three to five times daily, with either massage and passive joint movement three times daily or daily hydrotherapy) survived longer and maintained ambulation longer than patients who received moderate physiotherapy or no physiotherapy at all.^5^ Since the time of this publication, physiotherapy for dogs with DM has been widely recommended to, at the very least, improve the patient's quality of life.

Light or PhotoBioModulation therapy (PBMt) is an intervention currently used as an integral part of many rehabilitation protocols and to treat a variety of conditions in veterinary medicine.^9,10^ Since Mester's original publication (1968) demonstrated accelerated wound healing rates of skin incisions made to implant cancerous cells in rats,^11^ there have been many randomized, double-blind, placebo-controlled (RDBPC) studies of PBMt as a therapeutic agent with mixed results. At this time, the basic photochemistry and photobiology underlying the mechanism of action of PBMt have been sufficiently elucidated,^12–15^ thus, in this article we intentionally avoid discussion of the fundamental mechanism of action of PBMt; the interested reader is referred to the work by Hamblin and Demidova.^15^

The objective of this study was to describe the differences, if any, in the effects of two different PBMt protocols on the progression of DM clinical signs from their onset to nonambulatory paresis (NAP) or paralysis, and to euthanasia, and to compare their effects to existing historical data.

## Materials and Methods

### Screening process

**FIG. 1.**
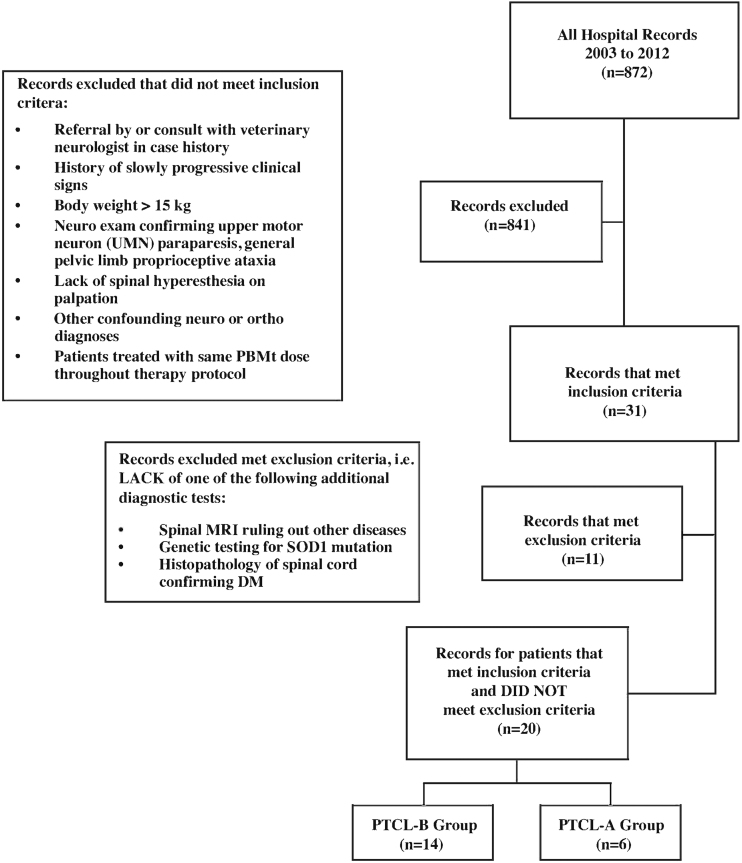
Flow chart for review of records and inclusion/exclusion criteria.

Authors collected all clinical records of dogs referred for presumed DM to a specialty physical rehabilitation facility in New England between 2003 and 2012. Owners of all dogs included in the study gave their permission for treatment before beginning both rehabilitation therapy and/or PBMt, the study did not require approval from any ethical committee as it is a retrospective review of clinical practice findings. Clinical records were screened for patients meeting the following *inclusion criteria*: patients were referred by a board-certified veterinary neurologist or a general practitioner who had consulted with a board-certified veterinary neurologist regarding the case before referral for rehabilitation therapy; patients with a history of slowly progressing clinical signs; patients with a body weight >33 lbs (15 kg) (medium to large breed dogs); patients scored on neurological examination confirming the presence of clinical signs consistent with stage I DM (as established by Coates and Wininger^2^) of UMN paraparesis, general pelvic limb proprioceptive ataxia, and lack of spinal hyperesthesia on palpation; patients with neurological and orthopedic examination ruling out the presence of other potential conditions confounding interpretation of clinical signs; and patients who were treated with the same PBMt dose (one laser device and treatment parameters) throughout the entirety of their rehabilitation therapy protocol. *Exclusion criteria*, records were excluded unless patients had one or more of the following diagnostics performed: magnetic resonance imaging (MRI) of the spine to rule out other lesions, DNA testing for *SOD1* mutation performed by Animal Molecular Genetics Laboratory (AMGL) at the University of Missouri, or postmortem histopathologic examination of the spinal cord confirming a diagnosis of DM. Twenty dogs met all the above inclusion and none of the exclusion criteria. These patients were sorted into the Protocol A (PTCL-A) group (*n* = 6) or the Protocol B (PTCL-B) group (*n* = 14) based on the PBMt protocol used to treat these patients throughout their course of therapy. The screening protocol is depicted in Fig. 1.

Authors reviewed three other study “cohorts” from the available published literature and considered them as “historical data” for general comparisons: Kathmann et al.,^5^ Polizopoulou et al.,^8^ and Kanazono et al.^16^ To the authors' knowledge, these are the *only* studies for which information on progression of ambulation status and/or survival data is reported for larger groups of dogs with DM. It is for this reason that even though the baseline characteristics of patients from each of these groups may have been different as well as the treatment interventions applied, they provide a set of historical “expectations” for disease progression and survival information. The comparisons are detailed in the **Results** and **Discussion** sections.

### Physical rehabilitation protocol

All dogs included in the review were prescribed twice weekly in-clinic rehabilitation therapy and an at-home exercise program. Occasionally, only one weekly visit was possible for some patients in either group. The in-clinic rehabilitation therapy was the same for all dogs and included PBMt, hydrotherapy exercise in an underwater treadmill, and other therapeutic exercises as outlined in Table 1. The at-home exercise program was also the same for all dogs and is also outlined in Table 1. In addition, all owners were asked to keep a log of patient daily activities. These logs were reviewed weekly with the owners throughout the course of therapy. In-clinic and at-home exercises were the same for both laser-treated groups. Supportive care recommendations were made including the use of assistive devices, such as slings for walking support, and protective boots, socks, or bandages for the feet, to be used as needed.

**Table 1.** Rehabilitation Therapy

In-clinic rehabilitation therapy (performed weekly to twice weekly)
Laser therapy (photobiomodulation therapy) as described in Table 2
Range of motion per limitations in patient evaluation
Stretching included hip extension and hip flexion with stifle extension
A hold of 15 sec on each, repeated three times for each stretch, and each limb
Controlled standing^a^ with correct paw placement on a mat until fatigue
Controlled standing^a^ with forelimbs on a balance disk (hind limbs are on a mat) to facilitate spinal extension, hip extension, core strength, and hind limb strength.
Controlled standing^a^ with hind limbs on a balance disk (forelimbs are on a mat) to facilitate core strength and hind limb strength
Controlled standing^a^ with hind limbs and forelimbs on one or two balance disks (depending upon the size of the dog)
Core work to the dogs' level: weight shifting in a standing, sitting, or down position for up to 5 min
Rhythmic stabilization—weight shifting back and forth in either position
Walking obstacles–cavaletti poles, or other objects requiring the dogs to lift their hind limbs up into flexion and encourage placement, performed until fatigue
Underwater treadmill—water height varies between level of the greater trochanter and stifle, speed at a walk and total walking time based on individual dog: all dogs started at 5 min duration at a walking pace at ∼1.0–2.0 mph. As they were able, they progressed. Typically, therapy would increase by 25% in time each week up to 30 min duration, as long as the patients tolerated everything well
*Home exercise program*
Controlled standing three times a day to tolerance with emphasis on correct paw placement and good footing
Controlled leash walking—three times a day for up to 15 min, assistance with a sling if needed
Controlled touching and massage—owners are encouraged to gently massage and touch their dogs' hind limbs to increase proprioception and awareness twice a day for at least 5 min
Includes stroking the legs against the hair, soft or light touch, thumping, and deep pressure
Walking backward—the dog is encouraged to walk backward throughout the day for a few steps if able

^a^ All of the controlled standing was performed until fatigue, at five repetitions.

**Table 2.** Protocol-A and Protocol-B Group Photobiomodulation Therapy Treatment Parameters

	PTCL-A group	PTCL-B group
Light parameters (dose)		
Wavelength (nm)	904	980
Radiant power (W)	0.5	6–12^a^
Irradiance (W/cm^2^) at skin surface	0.5	1.2–2.4^a^
Fluence (J/cm^2^)	8 (per “point”)	14–21 (average over treated area)
Treatment protocol	Point-to-point “grid method” technique at a total of 20 points spread throughout the treatment area according to manufacturer's instructions^b^	Continuously moving grid pattern over the entire treatment area at a speed of 1–3 in/sec according to manufacturer's recommendations^c^
Treatment area (cm^2^)	650–1000^d^	650–1000^d^
Treatment time	∼5 min, 20 sec	Between 25–26 min, 15 sec^a^

^a^ Depending on patient's size, larger patients treated at higher power; irradiance increased with increase in power.

^b^ Respond Model 2400XL Laser, Respond Systems, Inc., Branford, CT.

^c^ Companion Therapy Laser CTC-15, LiteCure, LLC, DE.

^d^ Treatment area increased with larger patient size.

PTCL-A, Protocol A; PTCL-B, Protocol B.

All dogs included in the review had PBMt performed noninvasively through the dog's coat; that is, the patients did not have their hair coat shaved as this is very often not practical (or permissible by pet owners) in a clinical setting. Treatment was applied with the laser probes in direct contact with the dog's coat and skin over the spinal column, as well as 5–7 cm lateral to the right and left sides of the spinal column in the paraspinal musculature, from approximately the T3 vertebral body to the lumbosacral junction. The PTCL-A and PTCL-B treatment groups are given in Table 2 below.

### Baseline demographics and disease progression

The baseline demographics for both groups: sex, hair coat length, body weight, age at treatment start, and time from onset of clinical symptoms to treatment start, were collected and described (Table 3**)**.

**Table 3.** Baseline Demographics

Patient	Sex	Body weight (lbs)	Haircoat length (1 = short, 2 = medium, 3 = long)	Age at StartTX (years)	Time from Sym Onset to StartTX (months)	Additional diagnostics^a^
PTCL-A group						
Chesapeake bay retriever 1	M	72	2	7.7	5.0	H
Chesapeake bay retriever 2	F	78	2	7.7	4.8	M^b^
Pit bull 1	F	49	1	8.3	5.6	M
Irish setter 1	F	68	3	6.8	3.3	M
Bernese mountain dog 1	M	108	3	7.2	3.5	H
Bernese mountain dog 2	M	97	3	9.9	3.3	H, G
Mean		78.67	2.33	7.93	4.25	
PTCL-B group						
German Shepherd 1	F	56	3	10.7	8.2	H
Mixed breed dog 1	F	44	3	7.2	7.8	M
Chesapeake Bay Retriever 3	M	62	2	5.7	8.3	G
Chesapeake Bay Retriever 4	F	78	2	7.3	5.7	G
Mixed breed dog 2	F	66	3	4	2.7	M
Pembroke Welsh Corgi 1	M	72	3	9.3	3.3	H
German Shepherd 2	F	52	3	6.1	7.6	M
Bernese Mountain Dog 3	F	78	3	6.2	5.1	G
Pembroke Welsh Corgi 2	M	47	3	8.4	22.5	M
Mixed breed dog 3	M	48	2	9.2	25.9	M
Chesapeake Bay Retriever 5	M	83	2	9.1	24.9	G
Bernese Mountain Dog 4	F	82	3	7.2	14.3	M
Chesapeake Bay Retriever 6	M	88	2	7.1	13.3	G
Mixed breed dog 4	M	33	3	8.1	6.7	G
Mean		63.5	2.64	7.53	11.15	

StartTX, time of treatment start; Sym Onset, onset of clinical signs.

^a^ Additional diagnostics: H, histopathology; M, MRI of spine; G, genetic testing.

^b^ All dogs listed, with the exception of one (Chesepeake Bay Retriever no. 2) had both TL and LS MRI performed. CBR no. 2 had only LS MRI performed.

LS, lumbosacral; MRI, magnetic resonance imaging; TL, thoracolumbar.

Additional individual diagnostics performed for each patient were reviewed and are also described in Table 3. Times of progression of clinical signs from their onset (Sym Onset) to NAP and to euthanasia, and from start of treatment to NAP and to euthanasia were collected and described (Table 4).

**Table 4.** Progression in Photobiomodulation Therapy Treated Groups

Patient	Time from Sym Onset to NAP (months)	Time from Sym Onset to Euth (months)	Time from StartTX to NAP (months)	Time from StartTX to Euth (months)
PTCL-A group				
Chesapeake Bay Retriever 1	10	13.34	4.98	8.36
Chesapeake Bay Retriever 2	7.34	13.02	4.82	8.2
Pit Bull 1	10.95	10.95	5.57	5.38
Irish Setter 1	7.02	7.02	3.34	3.67
Bernese Mountain Dog 1	7.87	8.82	3.51	5.31
Bernese Mountain Dog 2	9.54	13.41	3.34	10.07
Mean	8.79	11.09	4.26	6.83
PTCL-B group				
German Shepherd 1	31.2	33.82	22.98	25.61
Mixed breed dog 1	13.6	13.6	5.77	5.77
Chesapeake Bay Retriever 3	27.24	33.24	18.98	24.98
Chesapeake Bay Retriever 4	29.65	39.62	23.93	33.9
Mixed breed dog 2	9.12	9.12	6.43	6.43
Pembroke Welsh Corgi 1	35.3	49.11	31.97	45.77
German Shepherd 2	22.2	25.55	14.62	17.97
Bernese Mountain Dog 3	20.64	34.61	15.57	29.54
Pembroke Welsh Corgi 2	34.09	38.36	11.57	15.84
Mixed breed dog 3	47.19	54.24	21.31	28.36
Chesapeake Bay Retriever 5	49.35	56.93	24.49	32.07
Bernese Mountain Dog 4	38.87	50.87	24.59	36.59
Chesapeake Bay Retriever 6	36.57	42.67	23.31	29.41
Mixed breed dog 4	49.63	53.1	42.95	46.43
Mean	31.76	38.20	20.61	27.05

Euth, euthanasia; NAP, nonambulatory paresis.

### Statistical analysis

A multivariate analysis of variance (MANOVA) followed by a univariate comparison of means and a chi-square test examined the baseline demographics for the strength of their associations and statistically significant differences between the groups. Differences between the groups were deemed significant at α < 0.05.

The times of progression of clinical signs from their onset to NAP and to euthanasia, and from start of treatment to NAP and to euthanasia were compared using Cox Regression analysis controlling for sex, hair coat length, body weight, age at treatment start, and time from onset of clinical symptoms to start of treatment. Differences between the groups were deemed significant at α < 0.05. In addition, a Kaplan–Meier survival analysis was used to compare the elapsed time between onset of clinical symptoms and euthanasia for the PTCL-A and PTCL-B groups, and a single literature reference with sufficient detail to warrant the comparison—Polizopoulou et al.

## Results

A summary of the data collected is given in Tables 1–4:

Prescribed rehabilitation protocols, both at-clinic and at-home, given to both the PTCL-A and PTCL-B groups, are given in Table 1.

PBMt treatment protocols used to treat dogs in the PTCL-A and PTCL-B groups are detailed in Table 2.

Individual diagnostic tests and baseline demographic data: sex, hair coat length, body weight, age at treatment start, and time from onset of clinical symptoms to treatment start, are noted in Tables 3 and 4. None of the records reviewed showed a history of any other multisystemic diseases (hypoadrenocorticism, diabetes mellitus, cancer, etc.) that could account for a diminished life expectancy.

An initial MANOVA showed a significant difference in the noncategorical baseline demographic variables between the groups (*F*(4,15) = 203.522, *p* < 0.05). There was no difference in the categorical baseline variable (sex), χ^2^(1, *N* = 20) = 0.00, *p* = 1.0, that is, proportions of males and females were identical for the two groups. Follow-up univariate comparisons of the noncategorical baseline variables' individual means, within and not within the MANOVA context, showed the baseline difference to be significant for *only one* of the variables: time from onset of clinical symptoms to start of treatment. The mean time from onset of clinical symptoms to start of treatment for the PTCL-B group was significantly longer than that for the PTCL-A group (*F*(1,18) = 200.213, *p* = 0.05). Worth noting here that while the results that follow control for baseline differences (covariates), it would not normally be assumed that prolonging start of treatment (for any modality) from onset of clinical signs would be of any advantage.

Cox regression analysis controlling for differences in all demographic variables showed that the “survival” times were longer for the PTCL-B group.

The mean time between onset of clinical signs and NAP was significantly longer in the PTCL-B group (31.76 ± 12.53 months) than in the PTCL-A group (8.79 ± 1.60 months) (Wald statistics = 10.503, *p* < 0.05).

The mean time between onset of clinical signs and time of euthanasia was significantly longer in the PTCL-B group (38.2 ± 14.67 months) than in the PTCL-A group (11.09 ± 2.68 months) (Wald statistics = 10.747, *p* < 0.05). At the time of euthanasia, all dogs had progressed to Stage II DM and were nonambulatory paraparetic or paraplegic.

The mean time between start of treatment and NAP was significantly longer in the PTCL-B group (20.61 ± 9.81 months) than in the PTCL-A group (4.26 ± 0.98 months) (Wald statistics = 12.828, *p* < 0.05).

The mean time between start of treatment and time of euthanasia was significantly longer in the PTCL-B group (27.05 ± 12.39 months) than in the PTCL-A group (6.83 ± 2.41 months) (Wald statistics = 11.607, *p* < 0.05).

Although not of primary interest here, we note that in the analyses reported above, the relationship of survival to age at treatment start was positive and marginally significant (*p* < 0.10 for onset of clinical signs to NAP, *p* < 0.05 for onset of clinical signs to time of euthanasia, *p* < 0.10 for start of treatment to NAP, and *p* < 0.10 for start of treatment to time of euthanasia).

**FIG. 2.**
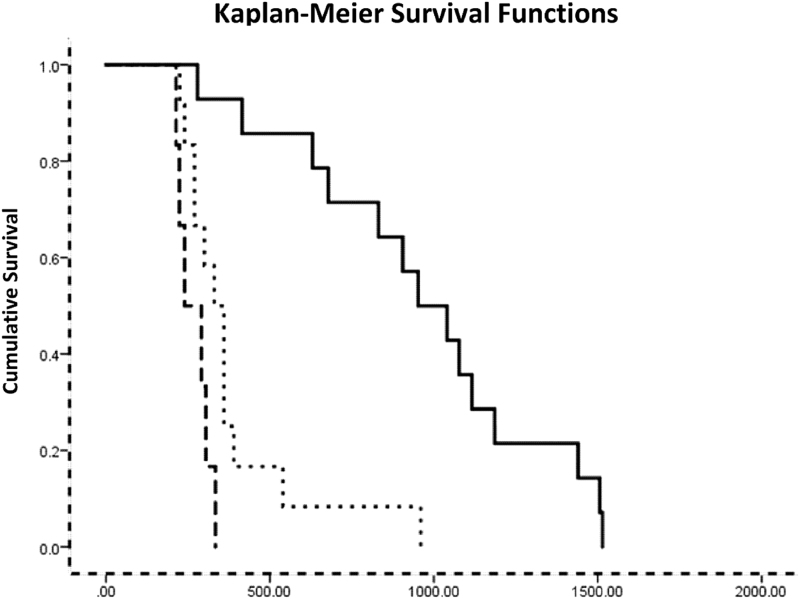
Survival functions for Protocol A group (PTCL-A, dashed line), Protocol B group (PTCL-B, solid line), and historical data.^8^

Kaplan–Meier survival analysis showed that the time from onset of clinical signs to NAP for the PTCL-B group was significantly longer than that of the PTCL-A group (Mantel-Cox Log Rank statistic = 20.434, *p* < 0.05) or the historical Polizopoulou et al. group (Mantel-Cox Log Rank statistic = 16.334, *p* < 0.05). The survival times for the PTCL-A and Polizopoulou et al.^8^ groups were similar (Fig. 2).

Historically, Kathmann's and Kanazono's reported data: mean survival time of 8.36 months (*n* = 9) and median time from Sym Onset to NAP of 10 months (*n* = 63), respectively, are very similar to those of the PTCL-A group: 11.09 months (*n* = 6), 8.79 months, respectively, and both much shorter than those of the PTCL-B group (*n* = 14): 38.2 months survival time and 31.76 months to nonambulation though no formal statistical comparisons could be made with these data sets.

## Discussion

The retrospective nature of this study, its small sample size, and the dearth of historical data from studies with similar parameters argue for caution with any conclusions from the data analysis results presented. Still, the analysis of the retrospectively collected data showed that the combination of PTCL-B PBMt and intensive rehabilitation therapy had a significant beneficial impact on the clinical symptom progression and survival times of dogs with a presumptive diagnosis of DM. This and the absence of effective treatments for DM, and potentially for people with ALS, warrant further investigation in a more controlled setting.

Antemortem diagnosis of DM can be difficult, largely because other acquired spinal cord diseases (intervertebral disk disease, lumbosacral stenosis, spinal cord neoplasia) or orthopedic issues (osteoarthritis in the pelvic limbs, cranial cruciate ligament injury in the stifle, etc.) can present with many similar clinical signs. In addition, it is not uncommon for older dogs with DM to also have one or more of these conditions. While a very thorough neurological and orthopedic examination may be able to differentiate some of the causes of these clinical signs, given the preponderance of other spinal cord diseases and orthopedic issues with similar clinical presentation, DM is largely diagnosed by ruling out these more common diseases as the cause of the progressive symptomatology previously outlined. This was one of the inclusion criteria for this study. At this time, a definitive diagnosis of DM can only be made by postmortem histopathologic examination of the spinal cord—showing characteristic axonal and myelin degeneration accompanied by increased astroglial proliferation in the most severely affected areas, usually the thoracic spinal cord.^1,17^ Other diagnostic tests may be undertaken, including neurodiagnostic imaging, electrodiagnostic testing, routine cerebrospinal fluid (CSF) analysis (all of which are normal in early DM), and genetic testing. Genetically, most DM dogs are homozygous for the SOD1: c.118G>A allele (though some dogs that are heterozygotes with no other SOD1 missense mutations will develop DM). While genetic tests may corroborate a diagnosis of DM, genetic screening alone is inadequate for diagnosis since SOD1 mutations are incompletely penetrant.^18^ However, in clinical practice, these further diagnostic tests may not be pursued for a variety of reasons, including access to resources and/or pet owner financial concerns. Given these limitations, the authors attempted to limit dogs in the study to only those that had at least one additional advanced diagnostic test result suggestive of DM (or at least ruling out other spinal cord disease in the case of spinal MRI). The lack of conclusive diagnosis (histopathology only, *n* = 5) for all dogs in the study is certainly a limitation and could have affected the results (i.e., some dogs might not have had DM), a prospective trial is needed to control for this limitation; however, the diagnostic limitations accurately reflect the clinical situation most often seen with veterinary patients suspected of having DM.

### Photobiomodulation parameters, light transmission, and differences in results between the groups

A basic axiom of PBM—the “Principle of Photochemical Activation” or Grothuss-Draper Law—states that only that light which is absorbed by a system can bring about a photochemical change.^19^ Thus, for transcutaneously delivered light to affect DM disease progression, the light's parameters and PBMt application protocols must be such that—after accounting for the light's energy losses in the coat, and the intervening tissues of the skin, muscle, etc.—a “sufficient” amount of light reaches and is absorbed by neural cells in the spinal cord. Work by Piao et al. provides a quantitative insight on the amount of light transmitted to the level of the spinal cord of cadaver dogs under safe, clinically acceptable skin-applied irradiances. Piao showed that there is a linear correlation between the irradiance of the light at the skin and that of the light measured at the spinal canal, and that on-contact surface irradiation results in higher transmission to the spinal canal in a large breed dog.^20,21^ Consequently, the irradiance at the spinal canal of patients in the PTCL-B group was between 2.4 and 4.8 times greater than that of patients in the PTCL-A group—1.2 to 2.4 versus 0.5 W/cm^2^ at the skin, respectively (Table 2). This estimate neglects differences in tissue transmission losses due to the different wavelengths used by the protocols: 904 nm in PTCL-A versus 980 nm in PTCL-B, as well as possible differences in therapeutic efficacy due to their different absorptions^22,23^ by cytochrome-c-oxidase. These differences are small compared with the differences in the protocol's skin-applied irradiances.

If it is in fact true, as suggested by the data analysis, that the combination of PBMt and intensive rehabilitation therapy delays the progression of clinical signs and extends the survival time of dogs with DM, the benefit is only true for patients in the PTCL-B group. It is possible that differences in musculoskeletal treatment benefits—due to differences in the protocols' PBM parameters—entirely account for the observed results. However, since the PBM parameters used in the PTCL-A group have been shown to be effective in musculoskeletal therapies,^24–28^ this is unlikely. A more plausible explanation is that the irradiance at the spinal cord delivered by the PTCL-B group was sufficient to produce a clinically meaningful effect, while that of the PTCL-A group was not. It is worth noting here that the laser used in Piao's studies was identical to the one used in the PTCL-B group in this retrospective analysis, and that the light's irradiance Piao measured at the spinal canal—shallowest region (T13-L1 region)—is comparable with that which was shown to be effective in two of three acute ischemic stroke trials in humans.^29–31^

### Possible mechanism of PBMt on disease progression

Although degenerative changes in DM occur throughout the spinal cord white matter, they are usually most severe in the middle to lower thoracic spinal cord segments in the dorsal portion of the lateral funiculus.^1,17,32^ This was the area where PBM treatment was applied for all patients included in the analysis.

The body of evidence in the literature has shown that the application of PBMt can effectively modulate the inflammatory response after not just peripheral nerve injury^33,34^ but in spinal cord injury (SCI) as well.^35,36^ Statistically significant suppression of proinflammatory cytokine and chemokine gene expression, significant decreases in the invasion of cells involved in secondary damage including macrophages/activated microglia and T lymphocytes, a reduction in astrogliosis, significantly improved axonal regrowth and function, and are all among the effects noted when PBMt is applied acutely after SCI.^36–38^ These studies provide evidence that light confers specific beneficial effects on the response of cells in the CNS to injury leading to alteration of the secondary injury response and progression of the injury process. Research involving transcranial PBMt (TPBMt) has taken these findings a step further and demonstrated significant improvements in neurological scores after ischemic stroke in laboratory animals,^39–41^ and in short- and long-term behavioral function as well as in reduction of brain tissue loss after traumatic brain injury.^41,42^ As mentioned previously in the discussion of light transmission, an effectiveness and safety trial on 660 human patients showed that TPBMt was safe with a trend toward efficacy for treatment of humans within 24 h of stroke onset.^29,30^

Numerous studies have suggested that astrocytes and the astrocyte glutamate transporter (GLT-1; via deficiency of glial reuptake of excitatory amino acids) may play a role in modifying disease progression and motor neuron (MN) loss in neurodegenerative disease progression.^43–47^ Astrocytes can induce MN degeneration through secretion of inflammatory mediators, including nitric oxide and prostaglandin E2,^48,49^ and at least one study has demonstrated that neural progenitor cell-derived astrocytes from both familial ALS (fALS) and sporadic ALS (sALS) patients were toxic to MNs *in vitro* by coculturing mouse embryonic stem-cell-derived MNs with the differentiated astrocytes from each patient. Increased proliferation of glial fibrillary acidic protein (GFAP)-positive astrocytes has been observed in the presence of atrophied MNs in the spinal cords of human patients with both fALS and sALS, and has been correlated with the severity of the axonal loss and demyelination in the spinal cords of dogs with DM.^46,47^ Ogawa et al. reported a significant and unique inducible nitric oxide synthase (iNOS) expression pattern (not induced by a T-cell-mediated pathway) in the gray matter of these same dogs, similar to that in SOD1 transgenic mice^50,51^ and in ALS patients.^52,53^ Citing previous reports that indicated that astrocytic nitric oxide production through iNOS is associated with glutamate uptake activity of GLT-1 or glutamate-induced excitotoxicity^54,55^ alongside the findings of decreased astrocytic GLT-1 expression in the spinal cord of DM dogs, Ogawa et al. concluded that there is likely an interaction between NO production and glutamate uptake occurring in DM.

The authors are aware of at least four studies reporting effects of PBMt on astrocytic activity. Yang et al. found that in astrocytes pretreated with PBMt, the typical Aβ-induced production of ROS and inflammatory response were eliminated in the cortical astrocytes of rats.^56^ PBMt has also demonstrated influence on the glial response, particularly that of the astrocytes, in a monkey model of Parkinson's disease.^57^ Sun et al. found that PBMt applied to the spinal cord of rats after SCI inhibited activation of GFAP-positive astrocytes as well as inhibited astrocyte proliferation and the expression of astrocyte activation-related genes *in vitro*.^58^ Finally, there is one published study specifically examining the use of PBMt in an SOD1 transgenic mouse model of ALS.^59^ This study reports that there was a statistically significant, yet short-lived improvement in the group that received laser therapy. The data suggest that there was a delay in the onset of motor deficits, but this beneficial effect was only seen in the early stage of the disease. The delay in onset of motor deficits in the early stage of the disease seen in the mice in this study is consistent with the suggestive results of the current retrospective analysis in dogs. Furthermore, there was a statistically significant decrease in GFAP expression (an astrocyte marker) in the cervical and lumbar enlargements of the spinal cord in the laser-treated groups of mice compared with controls. The authors concluded that laser may have suppressed astrocytes surrounding MNs in the spinal cord, possibly conferring a protective effect.

Further research is needed on astrocyte–neuronal interactions both before disease onset and during disease progression in DM as well on the effects of PBMt on these interactions; however, the authors suggest here that PBMt may be having a protective effect on the MNs in the spinal cord of DM dogs through similar mechanisms suggested in the studies mentioned above.

Besides PBMt having potential effects on the spinal cord, the authors do not discount an additional potential mechanism for the improvements in treated dogs noted in this study and that is as an ergogenic aid to therapeutic exercise and prevention to exercise induce muscle fatigue or damage. Recently, low-level laser (light) therapy has been used to increase muscle performance in intense exercises.^28,60^ Various studies have demonstrated the PBMt mediated increase in adenosine triphosphate in muscle as well as increase in the resistance of muscles to fatigue during intense exercise.^60,61^ Studies involving both mice and humans have demonstrated that patients treated with PBMt in various time frames before exercise are able to perform more repetitions in muscle fatigue tests and have improved muscle energy metabolism compared with control groups,^26,27,62–65^ and that the effect may be greater in older/elderly patients.^66^ Further studies have demonstrated that PBMt helps prevent muscle pain and soreness after excessive exercise, reduces muscle damage during exercise, facilitates postexercise recovery, and can also help heal muscle injuries through a variety of mechanisms.^25,60,67–69^ Studies that have been able to examine markers for oxidative stress and inflammation in muscle tissue from euthanized animals or in serum from other patients have demonstrated the PBMt accelerated or resolved the acute inflammatory response and reduced oxidative stress elicited by muscle trauma.^27,64,70^

Given the information included here, the authors propose one or more of the following possible mechanisms of action for PBMt in this study: a possible effect on the spinal cord, particularly a protective influence on the MNs in the early stages of the disease through a decrease in invasion of cells involved in secondary damage to the spinal cord or possible reduction in astrogliosis, a decrease in astrocytic nitric oxide production and subsequent influence on GLT-1, a restoration of healthy cellular energetics in the treated muscles along the spine, and/or a decrease in the muscle fatigue experienced by the treated patients aiding their therapeutic exercise program and assisting in recovery after exercise.

## Conclusions and Summary

### Potential impact

Absent any clinically tested, effective treatments for DM, clinicians usually pursue symptom palliation using conventional or novel therapies.^2^ Recently Endaravone was approved and granted orphan drug designation by the U.S. Food and Drug Administration (Press Announcement May 5, 2017; fda.gov), the first new treatment for ALS to receive FDA approval in many years. Edaravone is thought to confer neuroprotection in part through its free-radical scavenging activity.^71^ Although there have been studies looking at this drug in models of canine ischemia–reperfusion injury,^72,73^ there has not been any research yet specifically examining its potential use in treating DM. One recent study has investigated changes in endocannabinoid elements, which could serve as a first step in the development of cannabinoid therapy for both dogs with DM and possibly, people with ALS.^74^ Although this study was not prospective and limited by small sample size(s) and the inability to obtain postmortem histopathology on all dogs included in this study, it does suggest a possible beneficial effect of utilizing PTCL-B PBMt in combination with an intense rehabilitation therapy regimen. Absent effective therapies for DM, the challenges with preclinical trials for ALS, and the poor translation of success of novel neuroprotective therapeutics from small laboratory animal models to companion size animals and humans, PBMt combined with intense rehabilitation therapy should be considered for the palliative treatment of dogs suspected of being affected by DM, and may be an avenue for future research into therapy for humans with ALS.

### Prospective trial

Currently, the authors are planning a RDBPC trial to test PBM as a therapeutic for dogs with DM. The study will include a larger sample size, tighter inclusion criteria, placebo-controlled treatment with a sham laser device, as well as the same dosimetry used for both wavelengths of light mentioned here, 905 and 980 nm, and a more rigorous assessment of outcome. Ideally, this trial will be multicenter in nature, provided that each study site is capable of standardizing pre-enrollment diagnostics as well as treatment protocol, including rehabilitation therapy. Given the positive results of the current retrospective analysis, the possibility for a crossover design in dogs randomized to receive sham laser in this prospective study should be considered in the face of overwhelming lack of improvement and/or worsening of clinical signs after a given time. Ideally, laser calibration should be done regularly throughout the study period to guarantee that the proposed dose is the same as the delivered dose at the skin surface for any devices used during the entire study period.

## References

[1] Averill DR Degenerative myelopathy in the aging German Shepherd dog: clinical and pathologic findings. J Am Vet Med Assoc 1973;162:1045–1051 4196853

[2] Coates JR, Wininger FA Canine degenerative myelopathy. Vet Clin North Am Small Anim Pract 2010;40:929–950 20732599 10.1016/j.cvsm.2010.05.001

[3] Awano T, Johnson GS, Wade CM, et al. Genome-wide association analysis reveals a SOD1 mutation in canine degenerative myelopathy that resembles amyotrophic lateral sclerosis. Proc Natl Acad Sci USA 2009;106:2794–2799 19188595 10.1073/pnas.0812297106 PMC2634802

[4] Coates JR, March PA, Oglesbee M, et al. Clinical characterization of a familial degenerative myelopathy in Pembroke Welsh Corgi Dogs. J Vet Intern Med 2007;21:1323–1331 18196743 10.1892/07-059.1

[5] Kathmann I, Cizinauskas S, Doherr MG, Steffen F, Jaggy A Daily controlled physiotherapy increases survival time in dogs with suspected degenerative myelopathy. J Vet Intern Med 2006;20:927–932 16955818 10.1892/0891-6640(2006)20[927:dcpist]2.0.co;2

[6] Longhof SL, Duncan ID, Messing A A degenerative myelopathy in young German shepherd dogs. J Small Anim Pract 1990;31:199–203

[7] Clemmons RM Degenerative myelopathy. In: Current Veterinary Therapy X Small Animal Practice. Kirk RW (ed.). Philadelphia: W.B. Saunders Company, 1989; pp. 830–833.

[8] Polizopoulou Z, Koutinas A, Patsikas M, Soubasis N Evaluation of a proposed therapeutic protocol in 12 dogs with tentative degenerative myelopathy. Acta Vet Hung 2008;56:293–301 18828481 10.1556/AVet.56.2008.3.3

[9] Pryor B, Millis DL Therapeutic laser in veterinary medicine. Vet Clin North Am Small Anim Pract 2015;45:45–56 25432681 10.1016/j.cvsm.2014.09.003

[10] Sims C, Waldron R, Marcellin-Little DJ Rehabilitation and physical therapy for the neurologic patient. Vet Clin North Am Small Anim Pract 2015;45:123–143 25440754 10.1016/j.cvsm.2014.09.007

[11] Mester E, Szende B, Gärtner P The effect of laser beams on the growth of hair in mice. Radiobiol Radiother (Berl) 1968;9:621–626 5732466

[12] Karu TI Photobiological fundamentals of low-power laser therapy. IEEE J Quantum Electron 1987;23:1–17

[13] Karu TI Molecular mechanism of the therapeutic effect of low-intensity laser radiation. Lasers life Sci 1988;2:53–74

[14] Karu T Mitochondrial Mechanisms of Photobiomodulation in Context of New Data About Multiple Roles of ATP. Photomed Laser Surg 2010;28:159–160 20374017 10.1089/pho.2010.2789

[15] Hamblin MR, Demidova TN Mechanisms of low level light therapy. Proc SPIE 2006;6140:1–12

[16] Kanazono S, Pithua P, Johnson GC, et al. Clinical progression of canine degenerative myelopathy. ACVIM Forum Research Abstracts Program. J Vet Intern Med 2013;27:673–674

[17] Braund KG, Vandevelde M German Shepherd dog myelopathy—a morphologic and morphometric study. Am J Vet Res 1978;39:1309–1315 697138

[18] Toedebusch CM, Bachrach MD, Garcia VB, et al. Cerebrospinal fluid levels of phosphorylated neurofilament heavy as a diagnostic marker of canine degenerative myelopathy. J Vet Intern Med 2017;31:513–520 28186658 10.1111/jvim.14659 PMC5354061

[19] Calvert JG, Pitts JN Photochemistry. New York: Wiley & Sons, 1966

[20] Piao D, Sypniewski LA, Bartels KE Challenges of transcutaneous laser application for the potential of photobiomodulation of the spinal cord at the scale of a large companion animal. Proc SPIE 2017;10048:8–10

[21] Piao D, Sypniewski LA, Dugat D, Bailey C, Burba D, De Taboada L Transcutaneous transmission of photobiomodulation light to the spinal canal of dog as measured from cadaver dogs using a multi-channel intra-spinal probe. Lasers Med Sci 2019;34:1645–1654 30879228 10.1007/s10103-019-02761-0

[22] Jacques SL Optical properties of biological tissues: a review. Phys Med Biol 2013;58:5007–5008 10.1088/0031-9155/58/11/R37 23666068

[23] Mason MG, Nicholls P, Cooper CE Re-evaluation of the near infrared spectra of mitochondrial cytochrome c oxidase: implications for non invasive in vivo monitoring of tissues. Biochim Biophys Acta 2014;1837:1882–1891 25175349 10.1016/j.bbabio.2014.08.005 PMC4331044

[24] Bjordal JM, Couppé C, Chow RT, Tunér J, Ljunggren EA A systematic review of low level laser therapy with location-specific doses for pain from chronic joint disorders. Aust J Physiother 2003;49:107–116 12775206 10.1016/s0004-9514(14)60127-6

[25] Douris P, Southard V, Ferrigi R, et al. Effect of phototherapy on delayed onset muscle soreness. Photomed Laser Surg 2006;24:377–382 16875447 10.1089/pho.2006.24.377

[26] Leal ECP, Lopes-Martins RÁlB, Frigo L, et al. Effects of low-level laser therapy (LLLT) in the development of exercise-induced skeletal muscle fatigue and changes in biochemical markers related to postexercise recovery. J Orthop Sport Phys Ther 2010;40:524–532 10.2519/jospt.2010.3294 20436237

[27] De Marchi T, Leal ECP Jr, Bortoli C, Tomazoni SS, Lopes-Martins RÁB, Salvador M Low-level laser therapy (LLLT) in human progressive-intensity running: effects on exercise performance, skeletal muscle status, and oxidative stress. Lasers Med Sci 2012;27:231–236 21739259 10.1007/s10103-011-0955-5

[28] Ferraresi C, Huang Y-Y, Hamblin MR Photobiomodulation in human muscle tissue: an advantage in sports performance? J Biophotonics 2016;9:1273–1299 27874264 10.1002/jbio.201600176 PMC5167494

[29] Lampl Y, Zivin JA, Fisher M, et al. Infrared laser therapy for ischemic stroke: a new treatment strategy—results of the NeuroThera Effectiveness and Safety Trial-1 (NEST-1). Stroke 2007;38:1843–1849 17463313 10.1161/STROKEAHA.106.478230

[30] Zivin JA, Albers GW, Bornstein N, et al. Effectiveness and safety of transcranial laser therapy for acute ischemic stroke. Stroke 2009;40:1359–1364 19233936 10.1161/STROKEAHA.109.547547

[31] Stemer AB, Huisa BN, Zivin JA The evolution of transcranial laser therapy for acute ischemic stroke, including a pooled analysis of NEST-1 and NEST-2. Curr Cardiol Rep 2010;12:29–33 20425181 10.1007/s11886-009-0071-3 PMC2821619

[32] March PA, Coates JR, Abyad RJ, et al. Degenerative myelopathy in 18 Pembroke Welsh Corgi dogs. Vet Pathol 2009;46:241–250 19261635 10.1354/vp.46-2-241

[33] Hsieh Y-L, Chou L-W, Chang P-L, Yang C-C, Kao M-J, Hong C-Z Low-level laser therapy alleviates neuropathic pain and promotes function recovery in rats with chronic constriction injury: possible involvements in hypoxia-inducible factor 1α (HIF-1α). J Comp Neurol 2012;520:2903–2916 22351621 10.1002/cne.23072

[34] Masoumipoor M, Jameie SB, Janzadeh A, Nasirinezhad F, Soleimani M, Kerdary M Effects of 660- and 980-nm low-level laser therapy on neuropathic pain relief following chronic constriction injury in rat sciatic nerve. Lasers Med Sci 2014;29:1593–1598 24634001 10.1007/s10103-014-1552-1

[35] Anders JJ The potential of light therapy for central nervous system injury and disease. Photomed Laser Surg 2009;27:379–380 19569951 10.1089/pho.2009.0053

[36] Byrnes KR, Waynant RW, Ilev IK, et al. Light promotes regeneration and functional recovery and alters the immune response after spinal cord injury. Lasers Surg Med 2005;36:171–185 15704098 10.1002/lsm.20143

[37] Byrnes KR, Waynant RW, Ilev IK, et al. Genomic analysis of spinal cord following injury and photobiomodulation. Lasers Med Sci 2001;17:A28

[38] Byrnes KR, Waynant RW, Ilev IK, et al. Alteration in gene expression following spinal cord injury and photo-biomodulation. Lasers Surg Med 2003;Supp. 15:41

[39] De Taboada L, Ilic S, Leichliter-Martha S, Oron U, Oron A, and Streeter J Transcranial application of low-energy laser irradiation improves neurological deficits in rats following acute stroke. Lasers Surg Med 2006;38:70–73 16444697 10.1002/lsm.20256

[40] Lapchak PA, Wei J, and Zivin JA Transcranial infrared laser therapy improves clinical rating scores after embolic strokes in rabbits. Stroke 2004;35:1985–1988 15155955 10.1161/01.STR.0000131808.69640.b7

[41] Oron A, Oron U, Chen J, et al. Low-level laser therapy applied transcranially to rats after induction of stroke significantly reduces long-term neurological deficits. Stroke 2006;37:2620–2624 16946145 10.1161/01.STR.0000242775.14642.b8

[42] Oron A, Oron U, Streeter J, et al. Low-level laser therapy applied transcranially to mice following traumatic brain injury significantly reduces long-term neurological deficits. J Neurotrauma 2007;24:651–656 17439348 10.1089/neu.2006.0198

[43] Trotti D, Aoki M, Pasinelli P, et al. Amyotrophic lateral sclerosis-linked glutamate transporter mutant has impaired glutamate clearance capacity. J Biol Chem 2001;276:576–582 11031254 10.1074/jbc.M003779200

[44] Pardo AC, Wong V, Benson LM, et al. Loss of the astrocyte glutamate transporter GLT1 modifies disease in SOD1(G93A) mice. Exp Neurol 2006;201:120–130 16753145 10.1016/j.expneurol.2006.03.028

[45] Chien-Liang GL, Qiongman Kong GDC, Glicksman MA Glutamate transporter EAAT2: a new target for the treatment of neurodegenerative diseases. Future Med Chem 2012;4:1689–1700 22924507 10.4155/fmc.12.122 PMC3580837

[46] Ogawa M, Uchida K, Park E-S, et al. Immunohistochemical observation of canine degenerative myelopathy in two Pembroke Welsh Corgi dogs. J Vet Med Sci 2011;73:1275–1279. 1. 21628865 10.1292/jvms.11-0097

[47] Ogawa M, Uchida K, Yamato O, Inaba M, Uddin MM, Nakayama H Neuronal loss and decreased GLT-1 expression observed in the spinal cord of pembroke welsh corgi dogs with canine degenerative myelopathy. Vet Pathol 2014;51:591–602 23839236 10.1177/0300985813495899

[48] Hensley K, Abdel-Moaty H, Hunter J, et al. Primary glia expressing the G93A-SOD1 mutation present a neuroinflammatory phenotype and provide a cellular system for studies of glial inflammation. J Neuroinflammation 2006;3:2 16436205 10.1186/1742-2094-3-2 PMC1360663

[49] Haidet-Phillips AM, Hester ME, Miranda CJ, et al. Astrocytes from familial and sporadic ALS patients are toxic to motor neurons. Nat Biotechnol 2011;29:824–828 21832997 10.1038/nbt.1957 PMC3170425

[50] Almer G, Vukosavic S, Romero N, Przedborski S Inducible nitric oxide synthase up regulation in a trans- genic mouse model of familial amyotrophic lateral sclerosis. J Neurochem 1999;72:2415–2425 10349851 10.1046/j.1471-4159.1999.0722415.x

[51] Lee J, Ryu H, Kowall NW Differential regulation of neuronal and inducible nitric oxide synthase (NOS) in the spinal cord of mutant SOD1 (G93A) ALS mice. Biochem Biophys Res Commun 2009;387:202–206 19580782 10.1016/j.bbrc.2009.07.007 PMC2742676

[52] Phul RK, Shaw PJ, Ince PG, Smith ME Expression of nitric oxide synthase isoforms in spinal cord in amyotrophic lateral sclerosis. Amyotroph Lateral Scler 2000;1:259–267 10.1080/14660820050515089 11465019

[53] Sasaki S, Shibata N, Komori T, Iwata M iNOS and nitrotyrosine immunoreactivity in amyotrophic lateral sclerosis. Neurosci Lett 2000;291:44–48 10962150 10.1016/s0304-3940(00)01370-7

[54] Rao SD, Weiss JH Excitotoxic and oxidative cross-talk between motor neurons and glia in ALS pathogenesis. Trends Neurosci 2004;27:17–23 14698606 10.1016/j.tins.2003.11.001

[55] Ida T, Hara M, Nakamura Y, et al. Cytokine-induced enhancement of calcium-dependent glutamate release from astrocytes mediated by nitric oxide. Neurosci Lett 2008;432:232–236 18255223 10.1016/j.neulet.2007.12.047

[56] Yang X, Askarova S, Sheng W, et al. Low energy laser light (632.8 nm) suppresses amyloid-β peptide-induced oxidative and inflammatory responses in astrocytes. Neuroscience 2010;171:859–868 20884337 10.1016/j.neuroscience.2010.09.025 PMC2987533

[57] El Massri N, Moro C, Torres N, et al. Near-infrared light treatment reduces astrogliosis in MPTP-treated monkeys. Exp Brain Res 2016;234:3225–3232 27377070 10.1007/s00221-016-4720-7

[58] Sun J, Zhang J, Li K, Zheng Q, et al. Photobiomodulation therapy inhibit the activation and secretory of astrocytes by altering macrophage polarization. Cell Mol Neurobiol 2019; DOI: 10.1007/s10571-019-00728-x [Epub ahead of print] PMC11448964 31446561

[59] Moges H, Vasconcelos OM, Campbell WW, et al. Light therapy and supplementary Riboflavin in the SOD1 transgenic mouse model of familial amyotrophic lateral sclerosis (FALS). Lasers Surg Med 2009;41:52–59 19143012 10.1002/lsm.20732

[60] Hamblin MR Mechanisms and applications of the anti-inflammatory effects of photobiomodulation. AIMS Biophys 2017;4:337–361 28748217 10.3934/biophy.2017.3.337 PMC5523874

[61] Ferraresi C, de Sousa MVP, Huang Y-Y, Bagnato VS, Parizotto NA, Hamblin MR Time response of increases in ATP and muscle resistance to fatigue after low-level laser (light) therapy (LLLT) in mice. Lasers Med Sci 2015;30:1259–1267 25700769 10.1007/s10103-015-1723-8

[62] Lopes-Martins RAB, Marcos RL, Leonardo PS, et al. Effect of low-level laser (Ga-Al-As 655 nm) on skeletal muscle fatigue induced by electrical stimulation in rats. J Appl Physiol 2006;101:283–288 16627677 10.1152/japplphysiol.01318.2005

[63] Vieira WH, Ferraresi C, Perez SE, Baldissera V, Parizotto NA Effects of low-level laser therapy (808 nm) on isokinetic muscle performance of young women submitted to endurance training: a randomized controlled clinical trial. Lasers Med Sci 2012;27:497–504 21870127 10.1007/s10103-011-0984-0

[64] Liu X-G, Zhou Y-J, Liu TC-Y, Yuan J-Q Effects of low-level laser irradiation on rat skeletal muscle injury after eccentric exercise. Photomed Laser Surg 2009;27:863–869 19697999 10.1089/pho.2008.2443

[65] Levine D, DeTaboada L, Frydrych W, Dale RB Effects of laser on endurance of the rotator cuff muscles. Lasers Surg Med 2015;47(S26):44–45

[66] Larkin KA, Christou EA, Baweja HS, et al. Phototherapy Prolongs Time to Task Failure in Older Adults. New Orleans, LA: Society for Neuroscience, 2012

[67] Craig JA, Barron J, Walsh DM, Baxter GD Lack of effect of combined low intensity laser therapy/phototherapy (CLILT) on delayed onset muscle soreness in humans. Lasers Surg Med 1999;24:223–230 10229153 10.1002/(sici)1096-9101(1999)24:3<223::aid-lsm7>3.0.co;2-y

[68] Sussai DA, Carvalho P de TC de, Dourado DM, Belchior ACG, dos Reis FA, Pereira DM Low-level laser therapy attenuates creatine kinase levels and apoptosis during forced swimming in rats. Lasers Med Sci 2010;25:115–120 19554361 10.1007/s10103-009-0697-9

[69] Borsa PA, Larkin KA, True JM Does phototherapy enhance skeletal muscle contractile function and postexercise recovery? A systematic review. J Athl Train 2013;48:57–67 23672326 10.4085/1062-6050-48.1.12 PMC3554033

[70] Silveira PCL, Scheffer D da L, Glaser V, et al. Low-level laser therapy attenuates the acute inflammatory response induced by muscle traumatic injury. Free Radic Res 2016;50:503–513 26983894 10.3109/10715762.2016.1147649

[71] Sawada H Clinical efficacy of edaravone for the treatment of amyotrophic lateral sclerosis. Expert Opin Pharmacother 2017;18:735–738 28406335 10.1080/14656566.2017.1319937

[72] Sukmawan R, Yada T, Toyota E, et al. Edaravone preserves coronary microvascular endothelial function after ischemia/reperfusion on the beating canine heart in vivo. J Pharmacol Sci 2007;104:341–348 17721041 10.1254/jphs.fp0070186

[73] Xu J, Shen B, Li Y, et al. Edaravone attenuates ischemia-reperfusion injury by inhibiting oxidative stress in a canine lung transplantation model. Chin Med J (Engl). 2008;121:1583–1587 18982873

[74] Fernández-Trapero M, Espejo-Porras F, Rodríguez-Cueto C, et al. Upregulation of CB2 receptors in reactive astrocytes in canine degenerative myelopathy, a disease model of amyotrophic lateral sclerosis. Dis Model Mech 2017;10:551–558 28069688 10.1242/dmm.028373 PMC5451172

